# Malnutrition and Mortality Patterns among Internally Displaced and Non-Displaced Population Living in a Camp, a Village or a Town in Eastern Chad

**DOI:** 10.1371/journal.pone.0008077

**Published:** 2009-11-26

**Authors:** Gilles Guerrier, Malaïka Zounoun, Olimpia Delarosa, Isabelle Defourny, Michelo Lacharite, Vincent Brown, Biagio Pedalino

**Affiliations:** 1 Epicentre, Paris, France; 2 Medecins Sans Frontieres, Barcelona, Spain; 3 Medecins Sans Frontieres, Paris, France; AgroParisTech, France

## Abstract

**Background:**

Certain population groups have been rendered vulnerable in Chad because of displacement of more than 200,000 people over the last three years as a result of mass violence against civilians in the east of the country. The objective of the study was to assess mortality and nutritional patterns among displaced and non-displaced population living in camps, villages and a town in the Ouddaï and Salamat regions of Chad.

**Methodology:**

Between May and October 2007, two stage, 30-cluster household surveys were conducted among 43,900 internally displaced persons (IDPs) living in camps in Ouaddai region (n = 898 households), among 19,400 non-displaced persons (NDPs) living in 42 villages in Ouaddai region (n = 900 households) and among 17,000 NDPs living in a small town in Salamat region (n = 901 households). Data collection included anthropometric measurements, measles vaccination rates and retrospective mortality. Crude mortality rate (CMR), mortality rate among children younger than 5 years (U5MR), causes of death and the prevalence of wasting (weight-for-height z score <−2) among children aged 6 to 59 months were the main outcome measures.

**Conclusions:**

The CMR among the 4902 IDPs in Gozbeida camps, 4477 NDPs living in a village and 4073 NDPs living in a town surveyed was 1.8 (95% CI, 1.2–2.8), 0.3 (95% CI, 0.2–0.4), 0.3 (95% CI, 0.2–0.5) per 10,000 per day, respectively. The U5MR in a camp (n = 904), a village (n = 956) and a town (n = 901) was 4.1 (95% CI, 2.1–7.7), 0.5 (95% CI, 0.3–0.9) and 0.7 (95% CI, 0.4–1.4) per 10,000 per day, respectively. Diarrhoea was reported to be the main cause of death. Acute malnutrition rates (according to the WHO definition) among 904 IDP children, 956 NDPs children living in a village, 901 NDP children living in a town aged 6 to 59 months were 20.6% (95% CI, 17.9%–23.3%), 16.4% (95% CI, 14.0%–18.8%) and 10.1% (95% CI, 8.1%–12.2%) respectively. The study found a high mortality rate among IDPs and an elevated prevalence of wasting not only in IDP camps but also in villages located in the same region. The town-dweller population remains at risk of malnutrition. Appropriate contingency plans need to be made to ensure acceptable living standards for these populations.

## Introduction

Selecting a target population is a priority before implementing a food and relief aid program. Humanitarian aid agencies usually focus their effort on displaced population in emergency situations but pay little attention to the host population. Differences in outcome between residents and refugees have been reported in two studies but only after implementation of an intervention [1], [2]. There has been little research on the nutritional status and mortality among internally displaced and non displaced population prior to an intervention, particularly in Chad.

**Figure 1:**
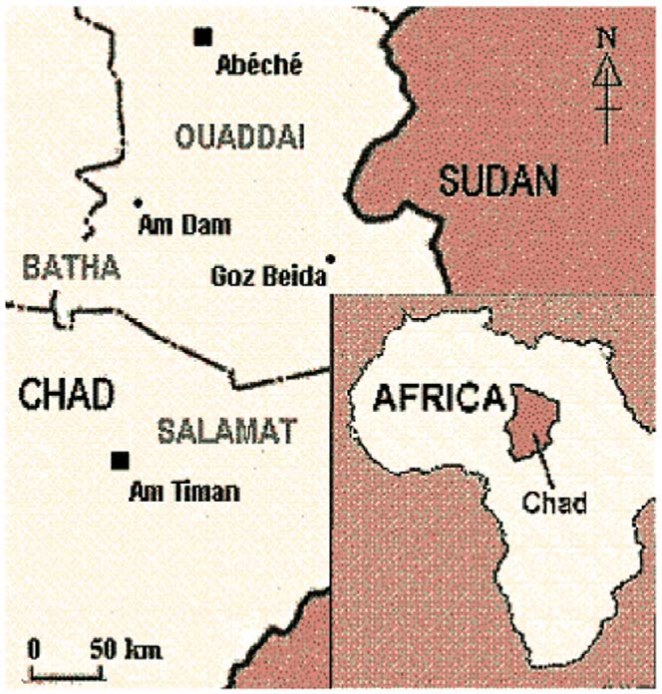
Location of Goz Beida, Am Dam and Am Timan in Eastern Chad.

Over the last three years, more than 150 000 internally displaced persons fled to south-eastern Chad as a consequence of inter-ethnic conflict. A first wave of attacks carried out in April 2006 resulted in the internal displacement of 50 000 people, mainly in Ouaddai region. A second period of attacks, which started in October 2006 displaced another 100 000 people in Salamat region. Generally, those vulnerable populations had lost all their assets and farms, if not their relatives. Recurrent food shortages between two harvests were possibly worsened by the resettlement of these displaced people. Médecins Sans Frontières (MSF) began work in eastern Chad in 2006 and operations targeting internally displaced people, including feeding centres and primary care clinics, opened in 2007, were focused on 3 areas: Goz Beida, Am Dam and Am Timan (Figure 1). Despite the international relief effort providing food, water, shelter and health care for displaced persons, nutritional security may have been compromised for villagers and town-dwellers surrounded by camps. We conducted a survey to estimate mortality rates and the prevalence of malnutrition in these three specific populations prior to any intervention.

## Methods

We undertook 2-stage, 30 cluster household consecutive surveys in three sites in Eastern Chad: one in camps welcoming internally displaced people (IDPs) (Goz Beida region), one in villages (Am Dam district) and one in a small town (Am Timan), both in areas surrounded by camps hosting IDPs.

In each area, a sample size of 900 children aged 6 to 59 months was required to achieve a 5% precision around an estimated prevalence of wasting of 15% with 95% confidence assuming a design effect of 2. With an estimated mean household size of 5 persons and with children younger than 5 years comprising 20% of the population, a total sample of 900 households, or 4500 persons, was required in each area.

The crude mortality rate (CMR) for Chad was estimated to be 0.5 per 10 000 per day (based on CMR observed in these regions). Assuming a cumulative mortality of 2% of the total population for the period of interest (recall period contained between 51 days and 6 month) and a design effect of 4 [3], a sample size of 5000 in each area would result in a precision of 2% with 95% confidence.

The sampling among IDPs in Goz Beida included 4 clearly identifiable camps (Koloma, Koubigou, Gouroukoun and Gassiré). Population size was estimated using a combination of reports of community leaders, UNHCR data [4] and shelter counts. In Am Timan town, data were obtained from local authorities. For these two areas, clusters were randomly selected using global positioning system coordinates following cluster allocation proportional to the population size of the camps.

The sampling frame included all villages in Am Dam district that had more than 40 households. Population data were obtained from chiefs of villages, and adjusted using estimates made by the MSF exploratory mission and by local health workers. In the first stage of the survey, 30 clusters were assigned proportionally to village population size. In the second stage, households were selected using standard immunization program methods [5]. At the centre of the village, a team member spun a pen to randomly choose a direction in which to conduct the survey. All houses in that direction were listed, counting from the centre to the periphery of the cluster and the first house to be surveyed was chosen by randomly choosing a number on the list and selecting the corresponding house.

A household was defined as a group of people who usually live under the same roof and share meals. If more than one household was present in the same dwelling, one was randomly selected. If an adult member was not at home at the time of the survey, the survey team returned to the household later in the day. If there was still no adult present, the next household was chosen. Subsequent households were selected by proximity (the next nearest household).

We used a standardized, pre-tested questionnaire for data collection. This survey instrument was tested in a non surveyed area, including training performed over three days at each site. Each survey team included a community health worker, a local person who spoke Arabic and French, and a member of the expatriate MSF staff who acted as a supervisor. The questionnaire was in French and the questions were asked in Arabic. Surveys included anthropometric measurements, measles vaccination history and retrospective mortality data collection. Each cluster was completed in one working day.

At each site, crude mortality rate, mortality rate among children younger than 5 years, prevalence of wasting (weight-for-height z score <−2) and vaccination status among children aged 6 to 59 months were assessed using within the same survey design. Because ages of children were not recalled reliably, the target age range of 6 to 59 months was substituted as a height of at least 65 cm and less than 110 cm. A standard United Nations Children's Fund (UNICEF) height board was used and children with a height of less than 85 cm were measured lying down. Weight was determined using a 25-kg Salter scale (UNICEF kit) that was calibrated daily. Acute malnutrition was defined according to standard weight-for-height z-score criteria or if there was pedal oedema.

A local event calendar was used to determine age and date of death. The total number of persons and children younger than 5 years present in each household was determined at the beginning of the survey in each site. Still births were counted neither as a live person nor a death. Neonates who had taken at least one breath after delivery were counted as dead.

The date most easily memorised among the surveyed populations was used to mark the beginning of the recall period. For Goz Beida and Am Dam, the celebration of the birth of the prophet Mohammed (Aïd el Mouloud) on March 30, 2007 was used, corresponding to a recall period of 51 days and 180 days, respectively. For Am Timan, the most applicable memorable date appeared to be the celebration of the beginning of the harvests on March 20, 2007, which gave a recall period of 207 days. Deaths in the household occurring among NDPs during the recall period were recorded. The calculation of the mortality rates was made using the current household census method. Inward migration to the sample households was assumed to be roughly equal to departures from those households. A series of structured questions were used to assign cause of death into categories based on World Health Organization case definitions [6]. Where corresponding local terms existed, such as diarrhoea, fever or malaria and respiratory tract infections, these were used to produce a less ambiguous classification. The questionnaire allowed other causes to be captured. Violence was not specifically asked about on the grounds that the surveyed areas were safe. Causes of death were recorded among NDPs but not among IDPs since a prospective mortality surveillance was scheduled in the camps.

### Ethical Considerations

All of the organizations involved in the survey subscribed to the ethical principles outlined in the Declaration of Helsinki [7]. Districts Leaders and local Chiefs gave permission to conduct the survey. The interviewee was the most senior adult household member, who gave oral informed consent to participate in the study. For children aged 6 to 59 months, consent to anthropometric measurement was obtained from a parent or a guardian. No incentives were offered to study participants. No names were obtained or recorded except when respondents agreed to the referral of malnourished children or sick individuals to the relevant clinics.

Data were analysed using EpiInfo software, version 6.04b (Center for Disease Control and Prevention, Atlanta, Ga) which includes C Sample for determining ninety-five percent confidence intervals for cluster surveys.

## Results

Surveys were performed from May 21–26, 2007, in Goz Beida, from October 10–17, 2007, in Am Timan, and from October 21–25, 2007, in Am Dam district. We surveyed 898, 901 and 900 households in IDP camps, NDPs in villages and NDPs in a town respectively. One household (0.1%) refused to take part in the survey in Am Dam district. Adults were not present in their households on the day of the survey in 10 cases (1.1%). The main characteristics of the surveyed population are described in Table 1. Overall, the number of reported deaths over the recall period in Goz Beida, Am Dam district and Am Timan were 45, 27 and 28 respectively. Of these, 23 (51%), 13 (48%), and 13 (46%) respectively were in children younger than 5 years. Among the 4902 IDPs, 4477 NDPs living in a village and 4073 NDPs living in a town surveyed during the period of interest, the CMRs were 1.8 (95% CI, 1.2–2.8), 0.3 (95% CI, 0.2–0.4), 0.3 (95% CI, 0.2–0.5) per 10 000 per day respectively and the respective mortality rates for children younger than 5 years were 4.1 (95% CI, 2.1–7.7), 0.5 (95% CI, 0.3–0.9) and 0.7 (95% CI, 0.4–1.4) per 10 000 per day. Diarrhoea was reported to be the leading cause of death among NDPs (Table 2).

**Table 1:** Survey profiles and mortality rates by age group and location in Ouaddai and Salamat regions.

Characteristics	Goz Beida, Ouaddai	Am Dam, Ouaddai	Am Timan, Salamat
	IDPs in camps	NDPs in villages	NDPs in a town
**No of households sampled**	898	901	900
**Population sampled, No**	4902	4477	4073
**Children<5 years, No. (%)**	1097 (22.4)	1158 (25.8)	905 (22.2)
**Household size, No. mean (range)**	5.5 (1–10)	5.0 (1–14)	4.5 (1–12)
**Recall period (days)**	51	180	205
**No. of deaths Total**	45	27	28
**No. of deaths <5 years**	23	13	13
**Mid-point population** *	4925	4436	4050
**CMR (95% CI)/Design effect**	1.8 (1.2–2.8)/2.2	0.3 (0.2–0.4)/1.9	0.3 (0.2–0.5)/1.8
**U5MR (95% CI)/Design effect**	4.1 (2.1–7.7)/2.1	0.5 (0.3–0.9)/2.0	0.7 (0.4–1.4)/1.9

Abbreviations: CI, confidence interval; IDP, internally displaced person; NDP, non-displaced person; CMR, crude mortality rate; U5MR, under five years old mortality rate.

* Mid point population = population at the time of the survey + ½[number of deaths-number of births].

**Table 2:** Causes of death among the surveyed population.

Causes of death	Goz Beida, Ouaddai	Am Dam, Ouaddai	Am Timan, Salamat	
	IDPs in camps	NDPs in villages	NDPs in a town	Total death among NDPs No (%)
**Diarrhoea**	Not reported	5	16	21 (38)
**Fever/Malaria**	Not reported	2	5	7 (13)
**Respiratory disease**	Not reported	6	1	7 (13)
**Unclassified death**	Not reported	14	6	20 (36)
**Total**	45	27	28	55 (100)

Acute malnutrition among 904 IDP children, 956 village NDP children, 901 town NDP children aged 6 to 59 months were 20.6% (95% CI, 17.9%–23.3%), 16.4% (95% CI, 14.0%–18.8%) and 10.1% (95% CI, 8.1%–12.2%) respectively (Table 3). Reported measles vaccination coverage ranged from 18% to 76% (Table 3).

**Table 3:** Childhood malnutrition and measles vaccination history among children aged 6 to 59 months alive at survey time by Eastern Chad.

Caracterisitcs	Ouaddai region	Ouaddai region	Salamat Region
	IDPs in camps	NDPs in villages	NDPs in a town
**Sample size, No.**	904	956	901
**Prevalence of malnutrition % (95% CI)/Design effect**	20.6 (17.9–23.3)/1.7	16.4 (14.0–18.8)/2.1	10.1 (8.1–12.2)/1.6
**Pedal oedema, No.**	0	1	4
**Measles vaccination history with card** * ** % (95% CI)**	71.0 (67.4–73.9)	3.4 (0.7–7.4)	8.0 (4.8–11.3)
**Measles vaccination history without card** ** ** % (95% CI)**	5.2 (3.9–6.9)	14.7 (5.0–24.5)	32.0 (23.8–40.1)

* Children vaccinated with a card available at survey time.

** Children vaccinated with no card available at survey time.

## Discussion

The prevalence of malnutrition was surprisingly high among NDPs living in villages, with more than 16% of children younger than 5 years being affected. This figure surpasses the 15% suggesting a very serious situation [8]. Our observation is even more alarming since the study was not performed at the peak season of acute malnutrition which is usually during the lean season (June to September) in similar areas of the Sahel [9]–[10]. However, comparisons with similar surveys carried out in the Sahelian zone should carefully be interpreted, since the effect of seasonality on malnutrition prevalence may differ from year to year, from country to country, and even from area to area [11]. Salamat region is considered the granary of Chad. The counter-season culture of berbere (a variety of sorghum) provides two harvests per year, creating a surplus that traditionally is exported to other regions including Ouaddai. This could explain the difference in the prevalence of malnutrition found between the two NDP populations. The 2006/2007 harvest was considered above average allowing exchanges of harvested cereals between the areas with surplus and those with shortages. Moreover, according to the National Rural Development Agency, productive rains were recorded in July and August, 2007, in the Sahelian zone of Chad. Producers were thus able to undertake agricultural activities. Despite this potential access to available food, several factors could explain our worrying results. Prices for food were higher than normal in the Ouaddai's regional capital, Abeche [12] partly due to disruptions in trade because of insecurity and heavy rainfalls rendering roads impassable. In addition, certain crops were threatened by pests such as grain-eating birds, which reduced harvests in the affected areas.

This extremely precarious situation may have been worsened by the surrounding IDPs seeking for assistance from NDPs. Moreover, the stationing of military troops nearby at the time of the survey probably contributed to a reduction in the food supply in the markets.

While the town-dweller population remained at risk of malnutrition, malnutrition was well above the threshold of 15% among under 5 year-old children in the IDP camps. This finding is consistent with a previous survey from another Sahelian region conducted among displaced populations [13]. Similar surveys conducted in Eastern Chad over the last trimester of 2008 showed an acute malnutrition level above 10% among children under five [14]. Such repeated high rates at different times suggest that the malnutrition among displaced populations in this region is chronic in nature. The impact of seasonality is therefore probably milder than described for non displaced populations. Even if IDPs are sometimes allocated a small piece of land to grow crops, their food security mainly relies on external relief action. This observation underlines the humanitarian problem which needs to be addressed.

The CMR in the IDP camps was higher than the 1.0 per 10 000 per day that is recognized internationally as defining an emergency situation [8], [15] while the CMR among NDPs population remains low, and is even below the expected rate in sub-Saharan populations. The main diseases found among the surveyed population did not differ substantially from the typical causes of mortality in the Sahelian zone such as respiratory diseases, malaria and diarrhoea. The under 5-year mortality rate exceeded the 2 per 10 000 per day used as the emergency benchmark in the IDP population. Unfortunately, the causes of death were not recorded in this area but diarrhoea was recorded to be a major cause of consultation among children in the primary care clinics [16], reflecting the lack of hygiene and difficult access to drinking water. This precarious situation worsened when waterborne diseases such as hepatitis E arose in the camps [16]. This finding underlines the necessity of considering improvement of living conditions and sanitation as a priority when intervening in these populations.

Despite recent vaccination campaigns, the coverage of measles vaccination remained insufficient in IDP camps (76.2%) and among NDPs living in the town (40.0%). The alarmingly low coverage (18.1%) among NDPs of villages outlines the logistical difficulties of vaccinating in remote districts with hard-to-reach populations. In view of this inadequate coverage, humanitarian and governmental agencies should be aware of the risk of measles outbreaks not only in IDP camps but also in the surrounding NDP populations.

This survey has a number of limitations including recall bias, a well known limitation in such retrospective mortality surveys [17], [18]. Moreover, since the recall period was different among the studied populations, there could be differential recall bias, particularly in the cases of Am Timan and Am Dam where longer recall period were used. Under-reporting of deaths, or erroneous reporting of their date were more likely to occurred due to forgetfulness, and may have subsequently led to an underestimate of mortality rates among NDPs. Establishing causes of death using a verbal autopsy is always challenging (with the exception of violence) and makes the validity of such data uncertain. Nevertheless, the use of trained field teams, of a rather short recall period (<1 year), of unambiguous cause of death categories and of a well recognised calendar date should have limited them. Under-reporting of particular situations related to local customs (e.g. the death of a child before 7 days of life) may have led to an underestimate of the mortality rate. We can not exclude the possibility that some residents may have been mixed with NDP population in the camps (when they were relatives or where the residents may have be intentionally increased the stated size of the camps in order to increase the quantity of food distributed). The snapshot view given by a nutritional prevalence survey which provides a picture at one point in time only is another limitation. Such results are extremely useful for planning immediate activities, but may be more difficult to interpret for relief agencies trying to develop more long term and sustainable projects, particularly in such complex situations.

This observational study found a high mortality rate among the displaced population but also a high prevalence of wasting not confined to IDP camps but also evident in villages located in the same region. Although there is no evidence that the displaced people contributed to a deterioration in the situation of the residents, our findings underline the potential impact that a displaced population might have on food security and health status in an already fragile host population. We recommend that aid programmes conduct assessment of the local populations welcoming refugees or displaced people before intervention in order to address this issue. Nutritional status, mortality rates as well as health care facility and drinking water access should be evaluated in these assessments.
